# Plasma filtration with dialysis (plasma diafiltration) in critically ill patients with acute liver failure

**DOI:** 10.1186/cc13589

**Published:** 2014-03-17

**Authors:** Y Eguchi, H Nakae, T Furuya, M Isono, Y Kishi, T Yoshioka

**Affiliations:** 1 Shiga University of Medical Science, Shiga, Japan; 2 Akita University School of Medicine, Akita, Japan; 3 Akita Red Cross Hospital, Akita, Japan; 4 Otsu Municipal Hospital, Shiga, Japan; 5 Hikone Municipal Hospital, Hikone, Japan; 6 Nishi-Kyoto Hospital, Kyoto, Japan

## Introduction

Removing the middle molecular weight substances including cytokines and albumin-bound toxin could be effective for patients with acute liver failure (ALF). We have developed a new system, plasma filtration with dialysis (plasma diafiltration (PDF)) [1],[2], and assessed its efficacy in multicenter analysis.

## Methods

A subgroup analysis of an observational study conducted in the ICUs of six hospitals. In PDF, simple plasma exchange is performed using a selective membrane plasma separator (Evaxclio EC-2A; Kawasumi Chemical Inc., Tokyo Japan), which has a sieving coefficient of 0.3 for albumin, while the dialysate flows outside the hollow fibers. The flow rate of the blood, dialysate, substitute and additional substitute was 80 to 100 ml/minute, 600 ml/hour, and 0 to 450 ml/hour according to the rate of water elimination and 150 ml/ hour, respectively. As the substitute from the additional fluid line, we added 1,200 ml (150 ml/hour) of fresh frozen plasma followed by 50 ml of 25% albumin considering the loss of albumin by diffusion. As an anticoagulant, nafamostat mesilate (Torii Pharmaceutical Co. Ltd, Tokyo, Japan) was used at a rate of 15 to 25 mg/hour.

## Results

A multicenter study was underway from October 2005 to August 2011. We performed PDF on 65 patients with ALF (severe sepsis, 22; post operation, 15; fulminant hepatitis, 11; alcohol hepatitis, 3; graft versus host disease, 4; and others, 10). The serum total bilirubin, plasma PT-INR and the model for end-stage liver disease (MELD) score before the PDF procedure were 15.0 ± 8.15 mg/dl (average ± SD), 2.3 ± 1.5 and 35.8 ± 9.3, respectively. PDF was performed as 9.2 ± 13.2 sessions per patient and the overall 28-day survival rate was 68.5%. According to the severity of the MELD score, we stratified patients into three categories defined by the MELD score. The numbers of patients were 15 (23%) in score 20 to 29, 30 (46%) in score 30 to 39 and 19 (29%) in score over 40. The 28-day survival rates were 73.3%, 40% and 16%, respectively.

## Conclusion

PDF may be a useful blood purification therapy for ALF, but PDF should be performed below a MELD score of 30.
